# Building healthier communities: effectiveness and cost-sustainability of the ASL3 GENESIS program for chronic diseases prevention

**DOI:** 10.1007/s40520-026-03429-w

**Published:** 2026-06-25

**Authors:** Luigi C. Bottaro, Enrico Torre, Andrea Giusti, Marta Caltabellotta, Isabella Cevasco, Dario Camellino, Gianni Testino, Simona Bottino, Sergio Di Matteo, Stefano Grego, Luca Degli Esposti

**Affiliations:** 1 General Direction, Azienda Sociosanitaria Ligure 3, Genoa, Italy; 2 Diabetes & Metabolic Diseases Unit, Azienda Sociosanitaria Ligure 3, Genoa, Italy; 3 Unit of Internal Medicine, Azienda Sociosanitaria Ligure 3, Genoa, Italy; 4 Health Direction, Azienda Sociosanitaria Ligure 3, Genoa, Italy; 5 Social & Health Direction, Azienda Sociosanitaria Ligure 3, Genoa, Italy; 6 Rheumatology Unit, Azienda Sociosanitaria Ligure 3, Genoa, Italy; 7 Hepatology Unit, Azienda Sociosanitaria Ligure 3, Genoa, Italy; 8 Territorial Pharmacy Unit, Azienda Sociosanitaria Ligure 3, Genoa, Italy; 9 S.A.V.E. Health Economics & Outcomes Research, Milan, Italy; 10 Budget & Accounting Unit, Azienda Sociosanitaria Ligure 3, Genoa, Italy; 11 CliCon S.R.L. Società Benefit, Bologna, Italy

**Keywords:** Population ageing, Chronic disease prevention, Integrated health-promotion programme, Heart failure, Chronic kidney disease, Chronic liver disease, Healthcare resource utilization, Economic sustainability

## Abstract

**Background:**

Population aging leads to rising chronic disease burden and healthcare costs. The metropolitan area of Genoa (Liguria), the oldest in Italy, is particularly affected. Since 2017, ASL3 has implemented a comprehensive health promotion strategy including risk-factor reduction initiatives and multidisciplinary care pathways for multimorbid patients.

**Aims:**

To evaluate the impact of the comprehensive health promotion program on the prevalence of heart failure (HF), chronic kidney disease (CKD), and chronic liver disease (CLD), and on healthcare resource use.

**Methods:**

Using the ASL3 data warehouse, we analyzed trends in HF, CKD, and CLD prevalence from 2011 to 2023 and compared yearly prevalence between 2011 and 2019 and 2020–2023. Hospitalization, outpatient activity and drug-prescription data were assessed; production and pharmaceutical costs were calculated. Regression models evaluated changes in prevalence trajectories.

**Results:**

The resident population and average age increased slightly (from 704,355 to 711,133 people; from 49 to 51 years old). The unadjusted and standardized prevalence of HF, CKD and CLD rose significantly over the study period. However, after regression analysis, significance was confirmed only for the period 2011–2019. In 2020–2023, prevalence continued to rise, but at a substantially slower rate, suggesting an attenuation of the upward trend.

**Conclusions:**

A multifaceted, system-wide prevention and care model may attenuate the upward trend in chronic-disease prevalence in highly aged populations. These findings support integrated health-promotion strategies as a sustainable approach to chronic disease management and resource stewardship.

**Supplementary Information:**

The online version contains supplementary material available at https://doi.org/10.1007/s40520-026-03429-w.

## Introduction

Japan and Italy present the oldest populations in the world, due to their low birth rates and increased longevity[1–3]. The inhabitants of the northern Region of Liguria show the highest mean age in Italy [2, 3]. Considering that the elderly population has a higher morbidity prevalence, our region, as well as our healthcare trust, which covers more than half of the regional population, represents an open-air laboratory for studying health-resource consumption in the ageing population.

Chronic conditions, in fact, worsen with age, and ageing itself increases the risk of multimorbidity. Among chronic diseases, heart failure (HF), chronic kidney disease (CKD), and chronic liver disease (CLD) represent highly prevalent and clinically relevant conditions in older adults, frequently co-occurring within complex multimorbidity patterns and contributing substantially to healthcare needs.

Multimorbidity, in turn, determines a larger economic burden on the health system and society as well. The structure of the health system and the offer of services and interventions determine how this burden is managed [4, 5]. The interplay between ageing and increased healthcare demand has been recognized early [6], and the intertwined nature of ageing, morbidity, and healthcare costs is well evident in the New Brunswick Health Council data [7]. In that report, the costs of sanitary care range from $1,266 per person/year with no chronic conditions, to $2,866 with one or two, and up to $5,232 in the presence of three or more, recognizing the importance of containing the progression of cumulative multimorbidity [7]. In this Canadian study, the annual average sanitary cost of subjects presenting three or more chronic conditions was four times that of individuals with none.

The most significant economic effect of multimorbidity is an increase in potentially preventable hospitalizations, and the total direct cost of these conditions is not purely additive but also depends on specific disease combinations and age groups [7]. In addition to the costs of hospitalizations, multimorbidity is also positively associated with total costs and care-transition costs, resulting in patients with up to three comorbidities having expected total costs that range between 1.55 and 2.85 times the mean expected total costs of subjects without any morbidity [8, 9].

All the above-mentioned highlights the need to emphasize the importance of prevention as well as early-diagnosis pathways, with the purpose of managing rising costs and improving health outcomes. Prevention must be implemented in order to avoid or delay the onset of chronic illness, whereas once chronicity has developed, it is mandatory to provide all the necessary care to ensure that no additional pathological conditions will be added in the near future, with its additional expenses and impact on quality of life [10].

Between 2017 and 2019, the Azienda Sociosanitaria Ligure 3 (ASL3) established a formal programme including population-based and subjects-at-risk interventions to reduce the burden of comorbid conditions in the metropolitan area of Genoa. This programme comprised several health-promotion and disease-prevention activities.

To assess the results of this programme, evaluate its efficacy and its economic impact and sustainability, ASL3 designed and implemented a retrospective observational ecological study, the GENESIS (GENoa Epidemiology for Sustainable health Improvement Study). The main objective of the GENESIS study was to analyze the impact of the health-promotion programme on the prevalence and temporal trends of chronic diseases and to investigate the effect on healthcare-resource utilization.

## Patients and methods

### Study setting

The metropolitan area of Genoa includes approximately 700,000 inhabitants and represents the oldest population in Italy. Healthcare services are coordinated by ASL3, which directly manages community, acute in-hospital (Villa Scassi Hospital, Padre Antero Micone Hospital, and Gallino Hospital), post-acute and chronic care, and collaborates with the others three acute hospitals in the metropolitan network (San Martino University Hospital, Evangelico Internazionale Hospital, Galliera Hospital). ASL3 maintains a centralized data warehouse integrating hospital discharges, outpatient services, drug prescriptions, general practitioner (GP) databases and exemption data for the metropolitan population. ASL3 is the unique healthcare entity that covers all medical activities in the metropolitan area of Genoa for the overall population.

### Health-promotion interventions and GENESIS framework

Between 2017 and 2019, ASL3 implemented a structured programme of population-based and high-risk interventions aimed at reducing the burden of comorbid conditions in the metropolitan area of Genoa. The programme was designed and coordinated by senior leadership and developed by a multidisciplinary team of public-health and clinical specialists, including health and social-health department directors, epidemiologists, nurses, and specialists in endocrinology, gastroenterology, rheumatology, nephrology, geriatrics, pulmonology, cardiology, neurology, gynecology, urology, psychiatry, orthopedics and other disciplines, depending on the specific project.

The main activities implemented included:

- A population-based healthy-lifestyle education programme promoted by the Hepatology Unit, aimed at reducing risk factors for chronic diseases and comorbidities, including harmful alcohol use, psychoactive substance use disorders, smoking, unhealthy diet, physical inactivity, sleep disturbances, social isolation, and problematic gaming/gambling behaviors;
- A comprehensive evidence-based smoking cessation programme, provided by the Cardiology Rehabilitation Unit;
- A systematic screening initiative for metabolic associated steatosic liver disease (MASLD) in the diabetic population of the metropolitan area, promoted by the Diabetes and Metabolic Diseases Unit, initially using shear-wave ultrasound elastography and subsequently upgraded with FibroScan^®^ technology for early detection of MASLD;
- The implementation of an outpatient cardiac rehabilitation programme for patients referred to ASL3 with established cardiovascular disease;
- An evidence-based diabetes-management programme promoted by the Diabetes and Metabolic Diseases Unit, implementing updated clinical practice guidelines and ensuring systematic prescription — in accordance with national regulations — of SGLT2 inhibitors and GLP-1 receptor agonists for patients with diabetes and established cardiovascular and/or chronic kidney disease;
- A telemedicine programme for multidisciplinary remote consultation, promoted by the Diabetes and Metabolic Diseases Unit, targeting individuals with multimorbidity (three or more chronic conditions) and involving specialist input from cardiologists, nephrologists, rheumatologists, geriatricians, and pulmonologists;
- A multifaceted system for the early recognition of autoimmune rheumatic diseases (AIRD), promoted by the Rheumatology Unit, employing fast-track pathways in emergency departments and primary-care settings to prevent disability and reduce the clinical burden associated with AIRD;
- A Cardio-Pulmo-Rheumatology joint clinic dedicated to patients with rare and/or complex diseases, providing a one-stop assessment with simultaneous evaluation by all three specialists;
- A comprehensive healthcare service dedicated to community-dwelling older adults, promoted by the Community Geriatric Unit, aimed at optimizing the management of frail and vulnerable individuals through early and integrated interventions based on the principles of Comprehensive Geriatric Assessment, with the aim of reducing multimorbidity burden, defining personalized diagnostic, therapeutic and rehabilitative pathways, preventing functional decline, promoting continuity of care, and addressing social isolation and socioeconomic vulnerability;
- The Ospivax Project, promoting the expansion of in-hospital and community vaccination units to improve adherence to national and regional immunisation programmes by removing logistical and cultural barriers to vaccination uptake in the general population and in individuals at high risk due to frailty and vulnerability (influenza and pneumococcal vaccination).

In addition to these initiatives, the ASL3 designed and initiated the GENESIS observational study to assess their effectiveness and associated resource utilisation, using the centralised ASL3 data warehouse.

### Data sources

An observational retrospective analysis was conducted using data from the administrative databases of ASL3, covering all health-assisted individuals in the metropolitan area of Genoa. These administrative datasets include comprehensive information on healthcare services and resources reimbursed by the Italian National Health Service (SSN). For this study, demographic, pharmaceutical, hospitalization, outpatient specialist care, and exemption databases were used.

The demographic database includes all demographic characteristics of individuals in the cohort. The pharmaceutical database contains data on all outpatient medications prescribed and reimbursed by the SSN, coded according to the Anatomical Therapeutic Chemical (ATC) system, including the number of packages dispensed, units per package, unit cost, and prescription date. The hospitalization database includes information on all hospital admissions, with discharge diagnoses classified according to the International Classification of Diseases, Ninth Revision, Clinical Modification (ICD-9-CM), and Diagnosis-Related Groups (DRG), along with DRG-related tariffs. The outpatient specialist services database includes data on diagnostic tests and specialist visits. Finally, the exemption database provides information on exemption type, code, and date.

All data were anonymized prior to analysis, and results were produced and reported exclusively in aggregated form. Ethical approval was obtained from the institutional ethics committee of the participating healthcare authority.

### Study population

All health-assisted individuals residing in the ASL3 catchment area between 2011 and 2023 were included in the analysis. Each subject was characterized annually according to demographic variables, including sex and age (reported as mean ± standard deviation). For each study year, the population was stratified by sex (male and female) and by age group (0–19, 20–29, 30–39, 40–49, 50–59, 60–69, 70–79, and ≥ 80 years).

### Selection of chronic diseases for analysis

The programme described above, which included population-based and high-risk interventions and was predominantly led by internal-medicine specialists, was designed to mitigate common risk factors for adverse clinical outcomes, including obesity, MASLD, hypertension, unhealthy diet, smoking, and physical inactivity. In this context, we hypothesized that these health-promotion activities would have an impact on the burden of three major chronic conditions: heart failure (HF), chronic kidney disease (CKD), and chronic liver disease (CLD).

### Chronic disease definition

HF, CKD, and CLD were identified through administrative coding (ICD-9-CM and exemption codes). HF was defined by the presence of at least one ICD-9-CM code 428 or the corresponding exemption code 021; CKD by ICD-9-CM code 585 or exemption code 023; and CLD by ICD-9-CM code 571 or exemption code 008.

### Prevalence analysis

Prevalence was defined as the number of individuals with a specific chronic condition per 1,000 persons in the corresponding cohort each year, calculated both overall and stratified by sex and age group. Age- and sex-standardized prevalence rates were calculated annually for each condition using the direct standardization method, with the 2023 Italian resident population as the reference. In this procedure, the age- and sex-specific prevalence estimates observed in the study cohort were applied to the corresponding strata of the reference population.

Linear regression models were used to evaluate annual changes in crude and standardized prevalence and to compare prevalence trends across two predefined periods: 2011–2019 and 2020–2023.

### Drug expenditure and healthcare costs analyses

For pharmaceutical expenditure, we analyzed nominal spending for the period 2015–2023. Nominal expenditure was defined as the total pharmaceutical spending reimbursed by the National Health Service (NHS). To convert nominal values into constant prices (expressed in 2023 euros), an annual deflator based on a price index (base 2023 = 100) was applied. Expenditure was also standardized for population ageing using a demographic adjustment coefficient, calculated as the ratio of the share of residents aged ≥ 65 years in each year to the corresponding share in 2023. Adjusted expenditure therefore reflects spending standardized for both inflation and demographic ageing.

Healthcare production costs of the ASL3 for the period 2017–2024 were also analyzed. Nominal expenditure referred to total annual production costs. To obtain constant-price values (expressed in 2024 euros), a price index (base 2024 = 100) was applied as a deflator. The consumer price index for blue- and white-collar households (FOI - *indice dei prezzi al consumo per Famiglie di Operai e Impiegati*), commonly used to derive GDP deflators in Italy, was used for this purpose.

Similarly, a demographic standardization coefficient was applied, calculated as the ratio of the ≥ 65 population share in 2024 to that of each respective year. Adjusted expenditure therefore reflects total costs standardized for both inflation and population ageing.

For pharmaceutical expenditure and healthcare production we could retrieve reliable and certified data respectively only since 2015 (pharmaceutical expenditure) and 2017 (healthcare production). This is the explanation of the three different periods of analysis.

## Results

The population of the metropolitan area of Genoa remained relatively stable between 2011 and 2023, ranging from 704,355 individuals in 2011 to 711,133 in 2023. The mean age increased from 48.8 ± 23.4 years to 50.9 ± 23.7 years over the same period, consistent with national and regional ageing trends [2, 3]. The proportion of older adults (≥ 50 years) rose, while the proportion of younger individuals (< 50 years) declined. Sex distribution remained stable, with males representing approximately 47–48% of the population throughout the study period (Table 1). Crude and standardized prevalence rates for HF, CKD and CLD are reported annually, stratified by sex and age group (Tables 2 and 3).

**Table 1:** Study population (metropolitan area of Genoa): overall, stratified by gender and study year, and stratified by age group and study year

	2011	2012	2013	2014	2015	2016	2017	2018	2019	2020	2021	2022	2023
**Total**	704,355	707,599	710,307	712,478	713,012	712,151	710,442	710,160	710,964	710,729	708,097	709,700	711,133
**Gender**													
Male	332,517	334,279	335,604	336,881	337,475	337,399	336,911	337,303	338,116	338,608	337,873	339,481	340,840
Female	371,838	373,320	374,703	375,597	375,537	374,752	373,531	372,857	372,848	372,121	370,224	370,219	370,293
**Age group**													
0–19	99,969	100,891	101,842	102,375	102,704	102,095	101,243	100,548	99,491	98,143	95,835	94,039	92,724
20–29	53,794	54,573	55,037	55,499	55,784	56,449	56,657	57,196	57,792	58,986	59,235	60,404	61,281
30–39	84,487	81,109	77,897	74,750	71,896	69,064	67,155	66,146	65,667	65,672	65,286	66,248	66,900
40–49	114,765	116,269	116,697	116,279	115,085	112,997	110,817	107,983	104,476	101,037	96,903	92,958	89,674
50–59	97,687	100,329	103,318	106,566	110,191	113,015	114,956	117,212	119,209	121,090	122,612	124,280	124,530
60–69	96,229	95,911	95,903	96,278	97,294	95,572	94,576	94,154	95,148	96,401	98,327	101,291	104,346
70–79	89,242	88,829	88,516	88,246	86,563	88,269	89,029	88,921	88,334	87,424	86,552	86,508	86,697
80+	68,182	69,688	71,097	72,485	73,495	74,690	76,009	78,000	80,847	81,976	83,347	83,972	84,981

**Table 2:** Gender-specific, overall and standardized prevalence x 1,000 of heart failure, chronic kidney disease and chronic liver disease among study participants by study year

		2011	2012	2013	2014	2015	2016	2017	2018	2019	2020	2021	2022	2023
Selected chronic disease	Gender													
Heart Failure	Male	10.0	11.1	12.3	13.1	14.0	14.6	15.0	15.5	16.1	15.3	15.4	15.7	16.4
	Female	8.8	10.1	11.1	12.2	12.7	13.0	13.5	13.8	14.6	14.0	14.2	14.2	14.6
	**Total**	9.3	10.6	11.7	12.6	13.3	13.8	14.2	14.6	15.3	14.6	14.8	14.9	15.5
	**Standardized**	9.4	10.6	11.7	12.6	13.3	13.8	14.2	14.6	15.3	14.6	14.8	14.9	15.5
Chronic Kidney Disease	Male	10.7	11.3	11.9	12.5	13.1	13.8	14.5	15.0	15.6	15.0	15.0	15.2	15.8
	Female	6.0	6.5	6.8	7.2	7.4	7.9	8.2	8.5	8.9	8.4	8.4	8.5	8.7
	**Total**	8.2	8.8	9.2	9.7	10.1	10.7	11.2	11.6	12.1	11.5	11.6	11.7	12.1
	**Standardized**	8.3	8.8	9.3	9.8	10.2	10.8	11.3	11.7	12.2	11.6	11.6	11.8	12.2
Chronic Liver Disease	Male	5.2	5.8	6.3	6.6	6.9	7.4	7.5	7.6	7.9	7.8	7.9	7.8	7.9
	Female	3.4	3.9	4.2	4.4	4.6	5.0	5.2	5.2	5.3	5.3	5.3	5.3	5.3
	**Total**	4.3	4.8	5.2	5.5	5.7	6.1	6.3	6.4	6.6	6.5	6.5	6.5	6.6
	**Standardized**	4.3	4.8	5.2	5.5	5.7	6.2	6.3	6.4	6.6	6.5	6.6	6.5	6.6

Standardization was performed using the direct method with the Italian population in 2023 as the standard population (see Table 1 and Table A1 in Appendix)

**Table 3:** Age-specific, overall and standardized prevalence x 1,000 of heart failure, chronic kidney diseases and chronic liver diseases among study participants by study year

Selected chronic disease	Age group	2011	2012	2013	2014	2015	2016	2017	2018	2019	2020	2021	2022	2023
Heart Failure	0–19	0.1	0.2	0.2	0.2	0.2	0.2	0.3	0.3	0.3	0.4	0.4	0.5	0.6
	20–29	0.2	0.3	0.3	0.3	0.4	0.4	0.4	0.4	0.4	0.5	0.5	0.4	0.4
	30–39	0.4	0.4	0.4	0.5	0.5	0.5	0.5	0.5	0.6	0.6	0.6	0.6	0.7
	40–49	0.9	1.0	1.1	1.2	1.1	1.1	1.2	1.2	1.3	1.2	1.2	1.1	1.2
	50–59	2.8	3.1	3.3	3.3	3.5	3.6	3.8	4.0	4.0	4.0	4.1	4.2	4.2
	60–69	8.5	9.4	10.5	11.1	11.6	12.1	11.9	11.9	12.1	11.9	11.8	11.6	11.9
	70–79	20.6	23.3	25.5	27.7	29.5	29.5	30.3	30.7	30.4	29.6	29.7	29.8	30.3
	80+	51.1	57.3	62.9	67.8	71.1	73.0	73.7	74.6	78.1	72.1	72.0	72.5	75.1
	**Total**	9.3	10.6	11.7	12.6	13.3	13.8	14.2	14.6	15.3	14.6	14.8	14.9	15.5
	**Standardized**	7.8	8.8	9.6	10.3	10.8	11.1	11.2	11.4	11.7	11.1	11.1	11.2	11.5
Chronic Kidney Disease	0–19	0.3	0.3	0.2	0.2	0.2	0.2	0.3	0.3	0.3	0.3	0.4	0.4	0.5
	20–29	0.7	0.7	0.9	0.9	0.9	0.9	0.9	1.0	1.0	1.0	0.9	0.8	0.8
	30–39	1.1	0.9	1.0	1.2	1.1	1.2	1.3	1.3	1.5	1.6	1.7	1.7	1.8
	40–49	1.9	2.1	2.1	2.1	2.2	2.2	2.2	2.3	2.3	2.3	2.3	2.3	2.5
	50–59	4.1	4.1	4.2	4.2	4.2	4.2	4.5	4.6	4.7	4.8	4.7	4.8	4.7
	60–69	8.6	9.4	9.6	9.8	10.2	10.8	10.7	10.8	10.7	10.3	10.6	10.7	10.9
	70–79	18.9	20.1	20.9	21.7	22.4	23.3	24.7	25.0	25.4	24.4	23.9	24.0	24.5
	80+	36.5	39.1	41.5	44.0	46.1	48.7	50.1	51.7	53.7	49.7	48.9	49.6	50.8
	**Total**	8.2	8.8	9.2	9.7	10.1	10.7	11.2	11.6	12.1	11.5	11.6	11.7	12.1
	**Standardized**	7.0	7.5	7.8	8.1	8.4	8.8	9.1	9.3	9.5	9.1	9.0	9.1	9.3
Chronic Liver Disease	0–19	0.3	0.4	0.4	0.4	0.4	0.4	0.4	0.4	0.4	0.4	0.5	0.6	0.7
	20–29	0.7	0.9	0.9	1.0	1.0	1.0	1.0	1.2	1.3	1.2	1.2	1.3	1.3
	30–39	1.4	1.7	2.0	1.9	2.1	2.3	2.2	2.1	2.2	2.0	1.9	1.9	1.8
	40–49	3.6	3.9	3.8	4.0	3.9	4.1	4.1	4.2	4.0	3.8	3.9	3.7	3.6
	50–59	6.6	7.3	7.9	7.9	8.2	8.4	8.5	8.6	8.5	8.1	7.8	7.6	7.4
	60–69	7.3	8.4	8.9	9.6	10.0	11.0	11.3	11.6	11.9	12.1	12.4	12.2	12.3
	70–79	7.7	8.5	9.3	9.9	10.7	11.4	11.6	11.3	11.9	11.6	11.8	11.9	12.5
	80+	5.2	5.9	7.0	7.5	8.0	8.6	8.9	9.2	9.8	9.7	9.7	9.4	9.5
	**Total**	4.3	4.8	5.2	5.5	5.7	6.1	6.3	6.4	6.6	6.5	6.5	6.5	6.6
	**Standardized**	4.0	4.5	4.9	5.1	5.3	5.6	5.7	5.8	5.9	5.8	5.8	5.8	5.8

Standardization was performed using the direct method with the Italian population in 2023 as the standard population (see Table 1 and Table A1 in Appendix)

Prevalence estimates increased significantly over the follow-up period across the overall population, among males and females, and within the older adult group (≥ 50 years) (Tables 2 and 3). In the overall population, the crude prevalence of HF (per 1,000 individuals) rose steadily from 9.3 in 2011 to 15.5 in 2023, indicating a sustained upward trend. Standardized prevalence also increased, although less markedly: from 9.4 to 15.5 in sex-standardized estimates (Table 2) and from 7.8 to 11.5 in age-standardized estimates (Table 3).

**Fig. 1:**
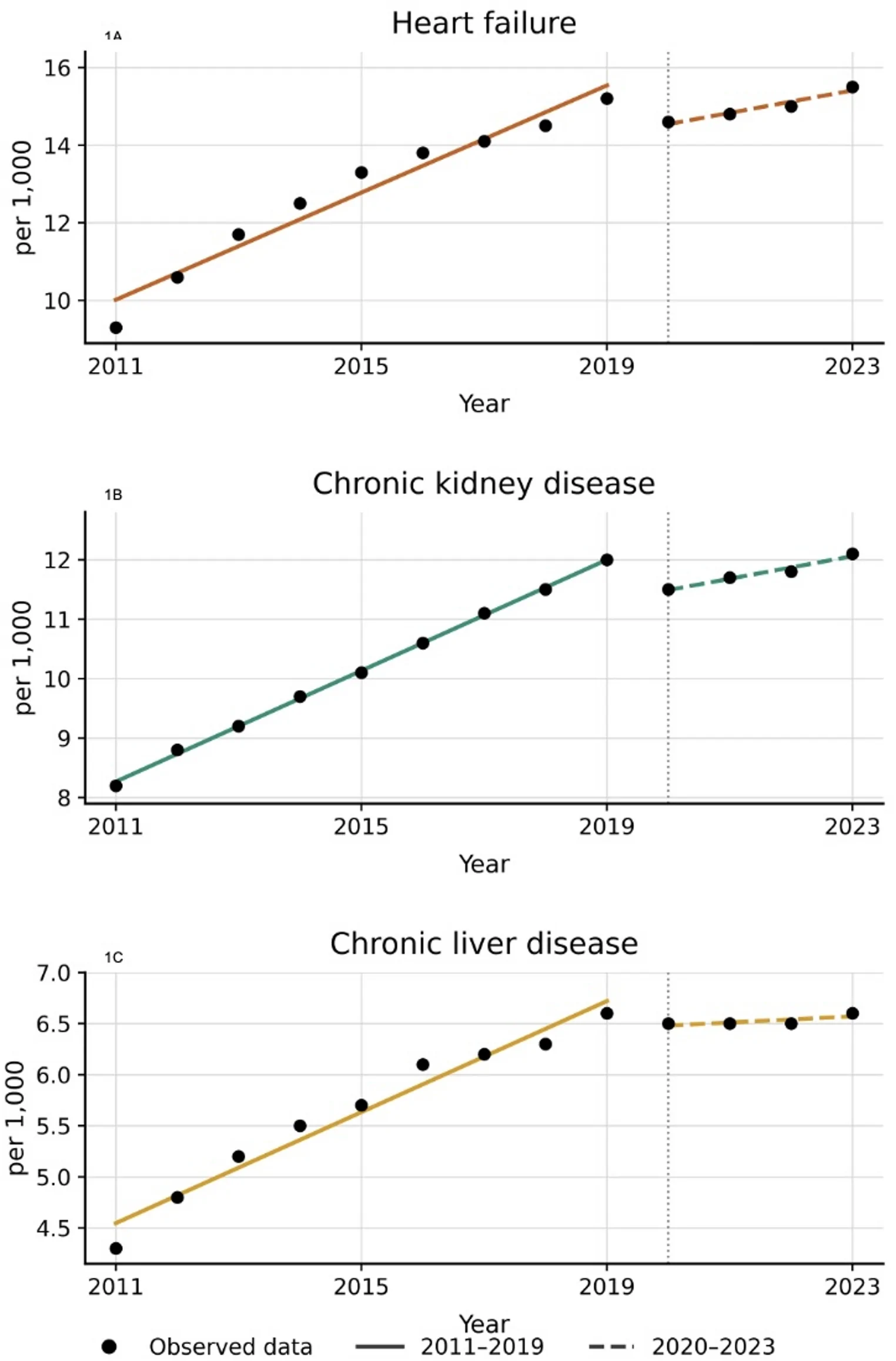
Crude annual prevalence (x 1,000) growth of heart failure (**A**), chronic kidney diseases (**B**), and chronic liver diseases (**C**) among study participants between years 2011–2019 and years 2020–2023

Linear regression analysis demonstrated a statistically significant increase of crude (+ 0.45/year) and age-standardized (+ 0.25/year) prevalence of HF from 2011 to 2023, as described in Table 4. Interestingly, when linear regression analysis was performed separately for two time periods (2011–2019 and 2020–2023), the increase of both crude and age-standardized prevalence remained significant only during 2011–2019, while no statistically significant increase was observed between 2020 and 2023 (Table 5; Fig. 1A), showing between 2020 and 2023 a less steep slope.

**Table 4:** Linear regression analysis of changes of crude and age-standardized prevalence of heart failure, chronic kidney disease and chronic liver disease between 2011 and 2023

Selected chronic disease	Slope	*P*-value	*R* ^2^
Heart Failure - Crude	+ 0.45/year	< 0.0001	0.84
Heart Failure - Standardized	+ 0.25/year	< 0.001	0.67
Chronic Kidney Disease - Crude	+ 0.32/year	< 0.0001	0.88
Chronic Kidney Disease - Standardized	+ 0.18/year	< 0.0001	0.77
Chronic Liver Disease - Crude	+ 0.18/year	< 0.0001	0.84
Chronic Liver Disease - Standardized	+ 0.13/year	< 0.0001	0.76

**Table 5:** Linear regression analysis of changes of crude and age-standardized prevalence of heart failure, chronic kidney disease and chronic liver disease between 2011–2019 and 2020–2023

Selected chronic disease	Years 2011–2019			Years 2020–2023		
	Slope	*P*-value	*R* ^2^	Slope	*P*-value	*R* ^2^
Heart Failure - Crude	+ 0.70/year	< 0.0001	0.96	+ 0.28/year	0.067	0.87
Heart Failure - Standardized	+ 0.46/year	< 0.0001	0.90	+ 0.13/year	0.113	0.79
Chronic Kidney Disease - Crude	+ 0.48/year	< 0.0001	1.00	+ 0.19/year	0.067	0.87
Chronic Kidney Disease - Standardized	+ 0.31/year	< 0.0001	0.99	+ 0.07/year	0.282	0.52
Chronic Liver Disease - Crude	+ 0.28/year	< 0.0001	0.97	+ 0.03/year	0.225	0.60
Chronic Liver Disease - Standardized	+ 0.23/year	< 0.0001	0.93	0.00/year	> 0.999	---

The crude and standardized prevalence of CKD showed a similar but less pronounced increasing trend compared to HF. The crude prevalence of CKD rose from 8.2 in 2011 to 12.1 in 2023. Corresponding sex-standardized prevalence were respectively 8.3 (2011) and 12.2 (2023), while age-standardized prevalence increased from 7.0 (2011) to 9.3 (2023). The mean annual increase in crude and age-standardized prevalence of CKD over the period 2011–2023 was statistically significant (Table 4), with crude prevalence rising by + 0.32/year and age-standardized prevalence by + 0.18/year. Similar to HF, when linear regression analysis was performed separately for the periods 2011–2019 and 2020–2023, the prevalence growth, both crude and age-standardized, between 2020 and 2023 was no longer statistically significant (Table 5; Fig. 1B), showing a mild slope.

Results for CLD demonstrated a similar but less pronounced growth trend, with a stabilization of prevalence increase observed between 2020 and 2023 (Tables 2 and 3). The crude prevalence of CLD increased from 4.3 in 2011 to 6.6 in 2023. Corresponding sex-standardized prevalences were identical, while age-standardized estimates showed a more modest increase, going from 4.0 (2011) to 5.8 (2023). Over the period 2011–2023, linear regression analysis indicated statistically significant annual increases in crude and age-standardized prevalence, with slopes of + 0.18/year and + 0.13/year, respectively (Table 4). When linear regression was performed separately for the periods 2011–2019 and 2020–2023), the mean annual increase in both crude and age-standardized prevalence was significant during 2011–2019, but showed a marked slowdown between 2020 and 2023 (Table 5; Fig. 1C).

Drug expenditure in the metropolitan area of Genoa from 2015 to 2023 and healthcare production costs from 2017 to 2024 are reported in Tables 6 and 7, respectively.

**Table 6:** Drug expenditure between 2015 and 2023 in the Metropolitan Area of Genoa

Year	Nominal Expenditure (€)	Price Index (base 2023 = 100)	Deflator (100/Index)	Expenditure at Constant 2023 Prices (€)	Population ≥ 65 years (%)	Standardization Coefficient (≥ 65_2023 / ≥65_year)	Adjusted Expenditure (€)
2015	136,530,668 €	83.68	1.20	163,154,148 €	21.7%	1.13	184,206,296 €
2016	136,135,283 €	84.52	1.18	161,130,112 €	22.0%	1.11	179,440,352 €
2017	135,755,894 €	85.36	1.17	159,947,452 €	22.3%	1.10	174,783,832 €
2018	137,707,842 €	86.28	1.16	159,012,872 €	22.6%	1.08	173,031,653 €
2019	142,781,893 €	87.03	1.15	164,061,887 €	22.9%	1.07	175,524,726 €
2020	148,199,435 €	86.69	1.15	170,984,329 €	23.2%	1.06	180,523,106 €
2021	150,147,226 €	87.87	1.14	170,882,640 €	23.4%	1.04	177,399,350 €
2022	161,162,277 €	94.98	1.05	169,681,869 €	24.0%	1.02	173,216,908 €
2023	168,705,502 €	100	1.00	168,705,502 €	24.5%	1.00	168,705,502 €

**Table 7:** Production costs between 2017 and 2024, referred to the ASL3

Year	Nominal Expenditure (€)	Price Index (base 2024 = 100)	Deflator (100/Index)	Expenditure at Constant 2024 Prices (€)	Population ≥ 65 years (%)	Standardization Coefficient (≥ 65_2024 / ≥65_year)	Adjusted Expenditure (€)
2017	1,165,090,183 €	84.35	1.19	1,381,156,034 €	22.6%	1.09	1,507,653,613 €
2018	1,163,092,834 €	85.28	1.17	1,364,866,457 €	22.8%	1.08	1,473,700,601 €
2019	1,173,879,575 €	85.70	1.17	1,369,681,694 €	23.1%	1.07	1,459,827,658 €
2020	1,718,849,077 €	85.45	1.17	1,371,427,025 €	23.4%	1.05	1,444,490,670 €
2021	1,151,020,656 €	87.07	1.15	1,324,398,335 €	23.7%	1.04	1,375,738,771 €
2022	1,191,649,944 €	94.12	1.06	1,266,047,033 €	23.9%	1.03	1,302,849,195 €
2023	1,231,477,128 €	99.21	1.01	1,243,328,945 €	24.2%	1.02	1,262,138,086 €
2024	1,325,424,243 €	100	1.00	1,325,424,243 €	24.6%	1.00	1,325,424,243 €

Nominal expenditure refers to total healthcare production costs of the Local Health Authority (ASL3). The price index (base 2024 = 100) was applied to obtain expenditure at constant 2024 prices through the corresponding deflator. The standardization coefficient adjusts for demographic ageing, calculated as the ratio between the ≥ 65 population share in 2024 and that of each year. Adjusted expenditure reflects total costs standardized for both inflation and ageing effects

Over the period 2015–2023 drug expenditure increased in nominal terms. However, after adjusting for inflation rate, the growth amounted to only 4%. Moreover, when the data were further standardized for the population aging coefficient, the trend revealed a relative decrease in drug expenditure, thus demonstrating, at least, the cost-effectiveness of the health promotion activities in regard of pharmaceutical expenditure.

The production costs during the period 2017–2024 exhibited a nominal growth of 13.8%, which declined to 4% after applying the FOI deflator. When additionally adjusted for the population aging coefficient, production costs showed an overall reduction of 12.1% over the same period.

## Discussion

To the best of our knowledge, this is the first published analysis describing the prevalence of three major disabling and resource-intensive chronic conditions—heart failure, chronic kidney disease, and chronic liver disease—in a well-defined metropolitan population in Italy. As detailed in the Methods, the highly structured organisation of the local healthcare system, together with the centralised data-management model under a single health authority (ASL3), covering the entire metropolitan area, provided a unique opportunity to evaluate temporal trends in disease prevalence and to assess the impact of a coordinated health-promotion programme.

In addition, the availability of administrative and economic data enabled us to estimate trends in drug expenditure and healthcare-production costs for the same population, allowing an initial assessment of economic impact and sustainability.

The key finding of this ecological observational analysis is the clear attenuation—and, in some cases, near plateauing—of disease-prevalence growth in the final years of observation (2020–2023), achieved alongside a reduction in direct healthcare costs, once adjusted for inflation and demographic aging. The likelihood that this trend is due to chance appears low, given the consistent flattening of the regression slopes over four consecutive years (Fig. 1), following the implementation of the ASL3 prevention programme. The temporal relationship between exposure and effect further supports biological and public-health plausibility.

Moreover, also the possibility that the above-described trend is due to COVID-19 pandemic is unlikely, since COVID-19 has increased chronic diseases instead of reducing them, as also recently suggested by a paper from Torre et al. [11].

Overall, our findings suggest that a multifaceted, system-wide prevention strategy can slow the growth of HF, CKD, and CLD prevalence, with a meaningful impact observable within three years of programme initiation.

Due to the ecological and observational design, causality cannot be inferred. The observed trends should therefore be interpreted as associations temporally coinciding with the programme implementation rather than direct causal effects.

Recent global analyses support our observations. Run J. et al. reported a deceleration in heart-disease prevalence growth from 2019 to 2021 in high-socio-demographic-index regions [12]. While prevalence continued to rise globally, high-income settings demonstrated a slowing trend comparable to ours. The authors attributed these effects to advances in HF management, including widespread uptake of SGLT2 inhibitors and optimisation of clinical-care pathways—strategies that were also central to our programme. We therefore believe that improvements in HF prevalence in our population are closely linked to evidence-based diabetes and cardiovascular management, lifestyle and smoking-cessation initiatives, cardiac rehabilitation, telemedicine for multimorbidity, and dedicated geriatric care models [12–14].

As with heart failure, evidence on temporal trends in chronic kidney and liver disease prevalence is limited, largely because repeated population-based assessments using consistent methodologies are required. In this context, available data on CKD prevalence are heterogeneous, with studies reporting increasing, stabilising, and even decreasing trends [15]. However, recent simulation studies project a continued rise in CKD prevalence over the coming years [16, 17]. For instance, Chertow et al. estimated a 6.3% increase in CKD prevalence in Italy between 2022 and 2027 [16].

In contrast to these projections, our findings show a clear attenuation of CKD-prevalence growth between 2020 and 2023, mirroring the trend observed for HF and suggesting that the programme may also have contributed to slowing CKD progression at the population level. This effect is plausibly linked to early and intensive management of diabetes and its complications through the use of SGLT2 inhibitors and GLP-1 receptor agonists, along with comprehensive geriatric services for community-dwelling older adults, among other programme components [12–14].

With the rising prevalence of obesity and diabetes, MASLD has become the leading cause of chronic liver disease, with its prevalence increasing markedly over the past three decades and expected to continue to rise [18, 19]. In addition, the COVID-19 pandemic negatively influenced alcohol-consumption patterns, potentially contributing to an increase in alcohol-related liver disease (ALD) [18]. These trends explain the growing burden of liver disease in Western countries.

In this context, two core components of our programme specifically targeted the principal modifiable risk factors for chronic liver disease—metabolic dysfunction and harmful alcohol use, which are key drivers of MASLD and ALD, respectively. The programme incorporated population-level and targeted interventions to reduce harmful alcohol consumption, along with strategies to mitigate metabolic risk through improved diabetes management (including GLP-1 receptor agonists) and promotion of healthy lifestyles [20].

Together with the broader suite of preventive actions, these measures were associated with a marked attenuation in CLD-prevalence growth, which reached a plateau between 2020 and 2023.

The findings on pharmaceutical expenditure and healthcare-production costs represent another key result of our study. After adjustment for inflation and population ageing, the programme demonstrated economic sustainability. Specifically, adjusted healthcare-production costs showed a significant decrease, which is likely attributable to a reduced clinical and economic burden associated with the three chronic diseases examined. This result is consistent with prior evidence indicating that innovative models of care can reduce adverse outcomes and ultimately lower healthcare expenditures by improving prevention and disease management [21, 22].

Our results on the trend in aggregate production costs, while encouraging, should be interpreted as an ecological association. They suggest a positive influence of the system program on overall efficiency, but they cannot establish a direct causal link or measure the impact of individual initiatives. However, these preliminary data strongly suggest the need for future dedicated studies, such as cost-effectiveness or cost-benefit analyses focused on the program, to assess its actual impact and sustainability.

In conclusion, this ecological analysis, which examined trends in heart failure, chronic kidney disease and chronic liver disease in a metropolitan health-planning context, suggests that the implementation of a structured, multifaceted prevention and care programme is both feasible and effective in slowing the growth of chronic-disease burden. These findings highlight the potential of integrated population-health strategies to improve clinical outcomes and promote long-term system sustainability.

## Supplementary Information

Below is the link to the electronic supplementary material.

Supplementary Material 1

## Data Availability

No datasets were generated or analysed during the current study.
