# Supplementary material for: Building healthier communities: effectiveness and cost-sustainability of the ASL3 GENESIS program for chronic diseases prevention
Source: Aging Clin Exp Res. 2026 Jun 25;38(1):186. doi: 10.1007/s40520-026-03429-w (PMC13558325; doi:10.1007/s40520-026-03429-w)
Supplement: Supplementary file 1 [file 40520_2026_3429_MOESM1_ESM.docx]

**Appendix**

**Table A1.** Italian population in 2023 by gender and age group.

| **Age classes** | **Male** | **Female** | **Total** |
| --- | --- | --- | --- |
| 0-19 | 5,276,413 | 4,962,794 | 10,239,207 |
| 20-29 | 3,092,371 | 2,841,643 | 5,934,014 |
| 30-39 | 3,341,745 | 3,259,041 | 6,600,786 |
| 40-49 | 4,126,798 | 4,150,743 | 8,277,541 |
| 50-59 | 4,728,610 | 4,873,456 | 9,602,066 |
| 60-69 | 3,730,673 | 4,033,129 | 7,763,802 |
| 70-79 | 2,796,155 | 3,276,960 | 6,073,115 |
| 80+ | 1,722,067 | 2,784,603 | 4,506,670 |
| **Tota**l | 28,814,832 | 30,182,369 | 58,997,201 |

**Source**: ISTAT (2023). Resident population. Available at: https://demo.istat.it/app/?l=it&a=2023&i=POS
